# A Case Report of Isolated Cuboid Nutcracker Fracture

**DOI:** 10.1155/2016/3264172

**Published:** 2016-05-26

**Authors:** Takaaki Ohmori, Shinichi Katsuo, Chiaki Sunayama, Katsunori Mizuno, Tomohiro Ojima, Kotaro Yamakado, Tomonari Ando, Shin Watanabe, Seigaku Hayashi, Hiroyuki Tsuchiya

**Affiliations:** ^1^Department of Orthopedic Surgery, Fukui General Hospital, 58-16-1 Egami, Fukui, Fukui 910-8561, Japan; ^2^Department of Orthopedic Surgery, Graduate School of Medical Science, Kanazawa University, Takaramachi 13-1, Kanazawa, Ishikawa 920-8641, Japan

## Abstract

Isolated cuboid fractures are very rare, since they typically occur in combination with midfoot fractures or dislocations. A 61-year-old man presented at our hospital with pain and swelling on the outside of his right foot. The lateral column of his right foot was shortened by approximately 6.5 mm on X-ray. CT showed displacement of the joint surface between the cuboid and the fourth metatarsal, with a 3.5 mm depression. An MRI revealed no other injuries. Based on these findings, we diagnosed the patient with an isolated nutcracker fracture of the cuboid. Using a 1.9 mm arthroscope, we examined the Lisfranc joint. Then the depressed fragments were elevated until the regular joint line was restored. A bone biopsy needle was then used to fill in the large defect with artificial bone. In this case, we did not plate the fracture. Six months after surgery, patient could walk without pain. We report a very rare case of isolated nutcracker fracture of the cuboid. In addition, we suggest our new treatment plan of this fracture.

## 1. Introduction

The cuboid is one of the most critical tarsal bones and constitutes a core element of both the longitudinal and transverse arch of the foot. The cuboid is the only bony structure supporting the lateral column of the midfoot [1], and it is required to maintain polyarticular alignment of the tarsal bones (the calcaneus, the lateral cuneiform, and the fourth and fifth metatarsals) [2]. Midfoot fractures are rare, with an annual incidence of 3.6/100000 in Edinburgh, and 50% of all midfoot fractures are cuboid fractures [1]. Isolated cuboid fractures are very rare, since they typically occur in combination with midfoot fractures (e.g., cuneiform or navicular) or dislocations (e.g., the Lisfranc joint).

Nutcracker fracture of the cuboid is a type of compression fracture that happens when severe abduction of the forefoot causes the cuboid to be caught between the base of the fourth and fifth metatarsals and the anterior surface of the calcaneus [3]. This is the first report of a case of isolated nutcracker fracture of the cuboid with a stable postoperative course.

## 2. Case Presentation

**Figure 1:**
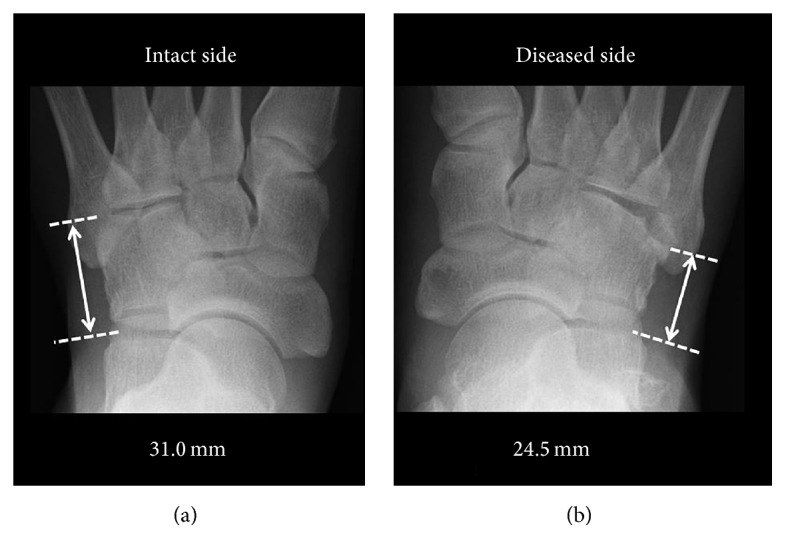
(a) Normal side with a lateral column of 31.0 mm. (b) Affected side with a lateral column of 24.5 mm.

**Figure 2:**
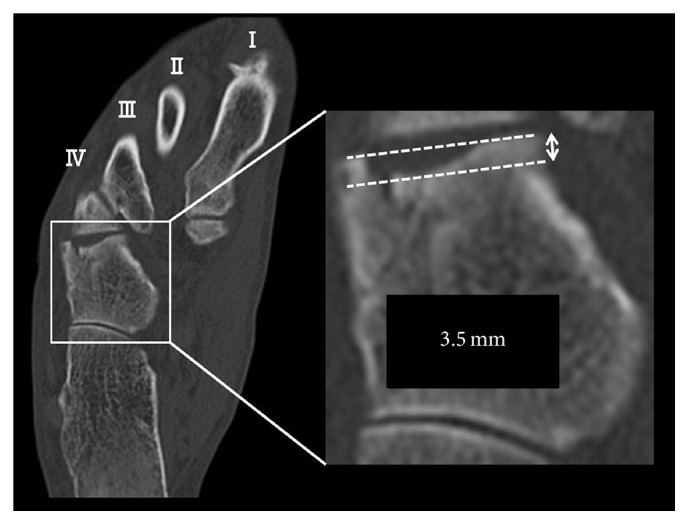
Depression of the cuboid articular surface between the cuboid and the fourth metatarsal (3.5 mm).

A 61-year-old man presented at our hospital with pain and swelling on the outside of his right foot. Right foot was injured falling into the groove of 50 cm during the work. He did not remember how his foot was twisted. The lateral column of his right foot was shortened by approximately 6.5 mm on X-ray (Figure 1). A computed tomography (CT) scan showed displacement of the joint surface between the cuboid and the fourth metatarsal, with a 3.5 mm depression observed in the range of the articular surface 3/4 (Figure 2). There are no other fractures except the cuboid. We took MRI to find other injuries. MRI showed only cuboid fracture. And there were no injuries of other bones and ligaments. There were no tenderness points except the cuboid. Based on these findings, we diagnosed the patient with an isolated nutcracker fracture of the cuboid and planned to perform an open reduction and bone grafting.

**Figure 3:**
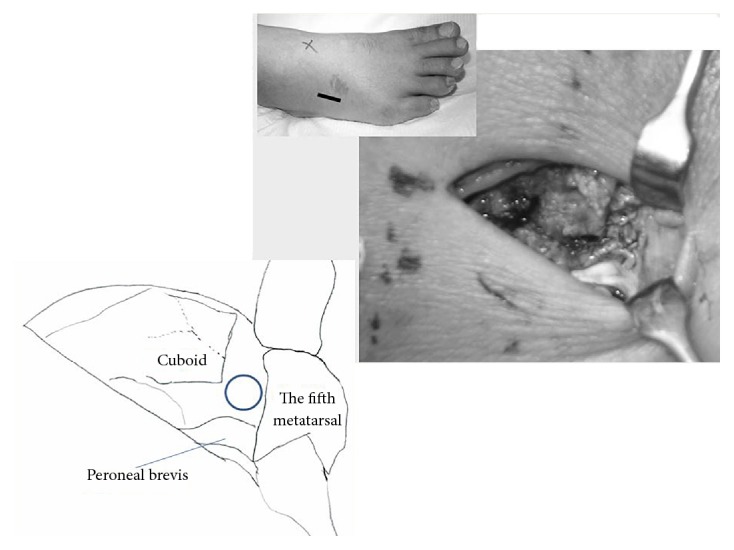
Intraoperative findings. ○: insert position of arthroscopy.

**Figure 4:**
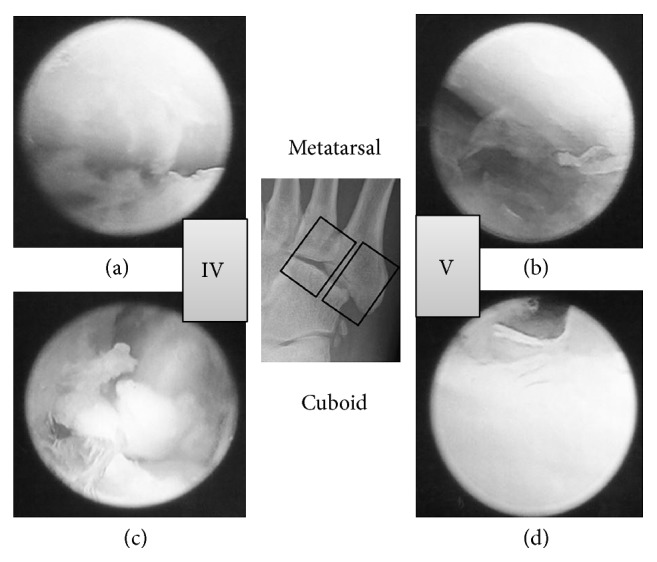
Arthroscopic findings. (a) Articular surface of metatarsal between the cuboid and the fourth metatarsal. (b) Articular surface of metatarsal between the cuboid and the fifth metatarsal. (c) Articular surface of cuboid between the cuboid and the fourth metatarsal. (d) Articular surface of cuboid between the cuboid and the fifth metatarsal.

**Figure 5:**
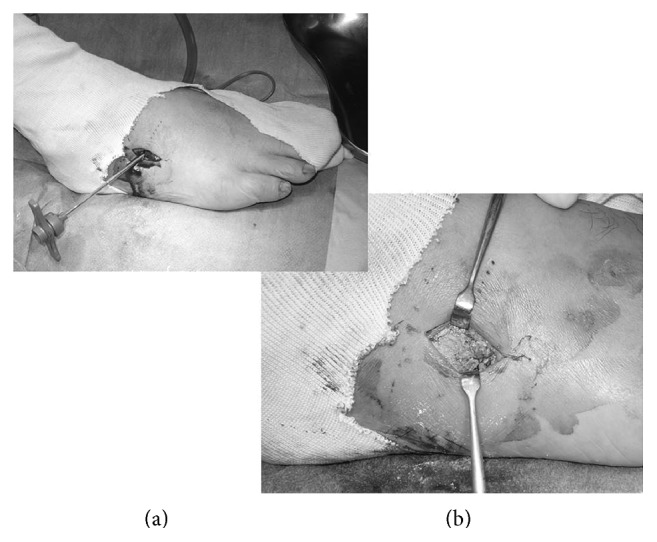
Intraoperative findings. (a) Artificial bone was applied into a large defect of the bone using a bone biopsy needle. (b) Appearance after the defect had been filled with artificial bone.

**Figure 6:**
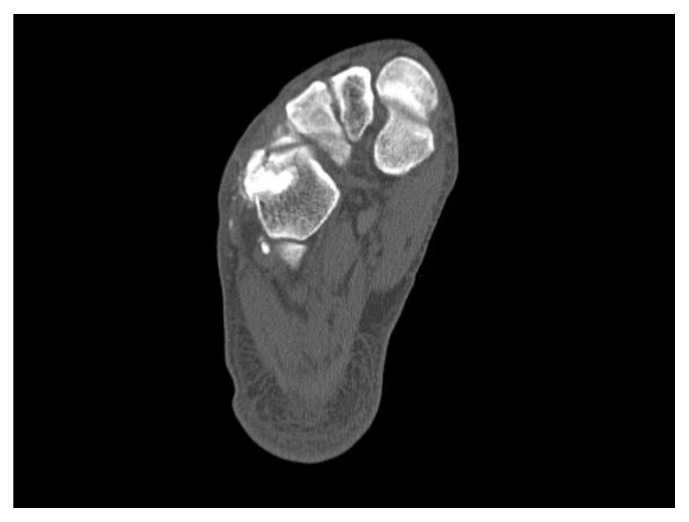
Defect area filled with artificial bone.

A lateral incision was made along the axis from the tip of the fibula to the tip of the fifth metatarsal. Next, we identified the Lisfranc joint using fluoroscopic guidance and made an incision in the joint capsule. Using a 1.9 mm arthroscope, we examined the Lisfranc joint (Figure 3) and noted that the cuboid articular surface between the cuboid and the fourth metatarsal was crushed (Figure 4). Articular surface of metatarsal was kept with a little fibrillation. The articular capsule was opened widely, and the fracture site was identified using fluoroscopic guidance. A hole was created at the fracture site, and the depressed fragments were elevated using surgical instruments, until the regular joint line was restored. A bone biopsy needle was then used to fill in the large defect with artificial bone (Figure 5). We used granular type OSferion® produced by OLYMPUS in Japan. We used about 2.0 g in the fracture site (Figure 6). Finally, the articular capsule was resutured.

The patient's right leg was placed in a cast that included the ankle joint. This nonweight-bearing cast was worn for 4 weeks, and then the patient began applying partial weight on the insole of his right foot to maintain the longitudinal and transverse arches. The patient walked without pain by postoperative week 8. Low-intensity pulsed ultrasound is said to promote bone union. So we used that device to promote bone union.

**Figure 7:**
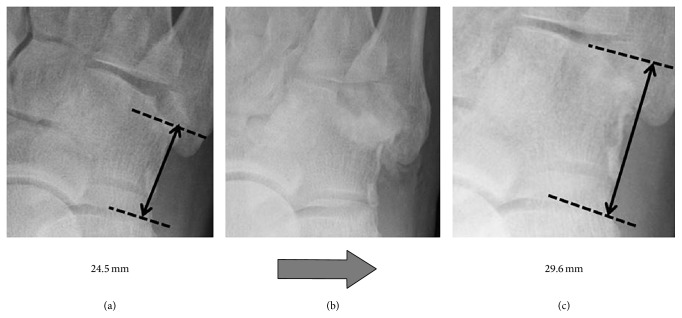
Lateral column length: (a) prior to surgery; (b) immediately after surgery; (c) 6 months after surgery.

**Figure 8:**
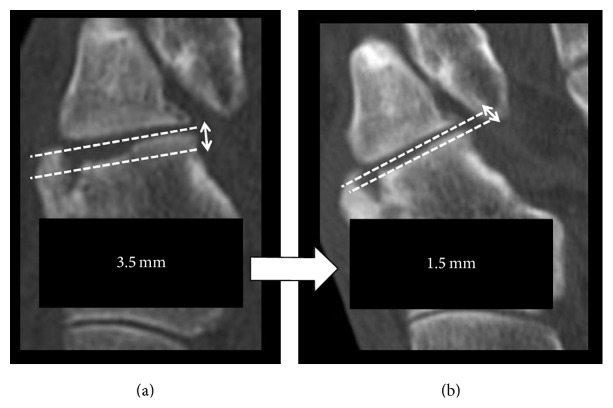
Depression of the cuboid articular surface: (a) prior to surgery; (b) 6 months after surgery.

Two months after surgery, the ROM of the ankle joint was still partially restricted, with 5° of dorsiflexion and 45° of plantar flexion. Six months after surgery, the lateral column of the patient's right foot was 29.6 mm in length on X-ray (Figure 7). The joint surface between the cuboid and the fourth metatarsal was depressed by 1.5 mm depression by CT scan (Figure 8).

The patient provided informed consent for the publication of this case report.

## 3. Discussion

There are no clear criteria for surgery for a nutcracker fracture. Hermel and Gershon-Cohen [4] recommended operation for fractures with comminution or dislocation. More recently, there has been a focus on lateral column shortening and joint surface displacement. Yu et al. [1] suggested that open reduction and fixation are indicated when either lateral column shortening or articular displacement exceeds 1 mm. Holbein et al. [5] suggested surgical repair only when the joint surface displacement is more than 1 mm or when the lateral column of the foot is shortened more than 3 mm. In the present case, we chose to operate as a best treatment course, since the articular displacement exceeded 3.5 mm, and the lateral column was shortened by more than 6.5 mm.

No operative procedure has been established for this injury. In most recent reports [1, 2], the treating physicians performed reconstructive surgery for lateral column shortening and articular displacement, followed by fixing the fracture with a miniplate or the calcaneocuboid joint with a plate and K-wire after bone grafting. In the present case, the distal fragment of the fracture was small, and we decided against the use of a plate. Ceroni et al. [6]. stated that internal fixation is not always necessary for children and adolescents, because the cuboid construct is often stable enough on its own, and the foot is protected by a plaster cast for weeks postoperatively. We did not find any reports in the literature in which internal fixation was not performed for an adult. We cannot explain the cuboid fracture type that do not require internal fixation. But cast for 8 weeks and low-intensity pulsed ultrasound could be adequate treatment plane for this fracture. It requires further consideration by increasing the number of cases on this point.

Most reports suggested immobilization for 6 weeks and not bearing weight for an additional 12 weeks. But in this case, we selected short postoperative rehabilitation program. Six months after surgery, his right lateral column had lengthened by approximately 5 mm, and the articular surface between the cuboid and the fourth metatarsal expanded by approximately 2 mm. Compared with his left foot, there was still some shortening of the right lateral column and joint depression, but his range of motion had improved and he could walk without pain.

In this way we avoided fixing the tarsometatarsal joint and calcaneocuboid joint as reported by Yu et al. [1]. Although the patient's right foot lateral column and joint line were stable 6 months after surgery, long-term follow-up is still required.

We report a very rare case of isolated nutcracker fracture of the cuboid. In most cases of nutcracker fracture, sprained ankle would be happening at the same time. Therefore, other injuries are also merged. I think that this case is caused by only valgus force of the forefoot. It is very rare case. An operation was performed to reconstruct the crushed bone and prevent possible joint destruction. A satisfactory outcome was achieved despite performing not only internal fixation but also short postoperative rehabilitation program.
